# Regulatory interfaces surrounding the growing field of additive manufacturing of medical devices and biologic products

**DOI:** 10.1017/cts.2018.331

**Published:** 2018-11-29

**Authors:** Joan E. Adamo, Warren L. Grayson, Heather Hatcher, Jennifer Swanton Brown, Andrika Thomas, Scott Hollister, Scott J. Steele

**Affiliations:** 1 Clinical & Translational Science Institute, Biomedical Engineering Department, University of Rochester Medical Center, Rochester, NY, USA; 2 Translational Tissue Engineering Center, Johns Hopkins University School of Medicine, Baltimore, MD, USA; 3 Womble Bond Dickinson (US) LLP, Winston-Salem, NC, USA; 4 Spectrum, Stanford Center for Clinical & Translational Research & Education, Stanford, CA, USA; 5 Core Laboratory of the Clinical & Translational Science Center, Weill Cornell Medicine, New York, NY, USA; 6 Center for 3D Medical Fabrication and Department of Biomedical Engineering, Georgia Institute of Technology/Emory University, Atlanta, GA, USA; 7 Clinical & Translational Science Institute, Department of Public Health Science, University of Rochester Medical Center, Rochester, New York, NY, USA

**Keywords:** Regulatory science, additive manufacturing, 3D printing, precision medicine, FDA

## Abstract

Rapidly advancing technology often pulls the regulatory field along as it evolves to incorporate new concepts, better tools, and more finely honed equipment. When the area impacted by the technological advancement is regulated by the Food and Drug Administration (FDA), a gap develops between the technology and the guidelines that govern its application. Subsequently, there are challenges in determining appropriate regulatory pathways for evolving products at the initial research and developmental stages. Myriad factors necessitate several rounds of iterative review and the involvement of multiple divisions within the FDA. To better understand the regulatory science issues roiling around the area of additive manufacturing of medical products, a group of experts, led by a Clinical and Translational Science Award working group, convened the Regulatory Science to Advance Precision Medicine at the Fall Forum to discuss some of the current regulatory science roadblocks.

## Introduction

Additive manufacturing, also referred to as three-dimensional (3D) printing, holds tremendous promise as a precision medicine tool to effectively customize treatments to individuals [1–4]. As this technology advances, the regulatory field surrounding its clinical, diagnostic, or therapeutic use is also evolving. Regulatory science is defined by the Food and Drug Administration (FDA) as “the science of developing new tools, standards, and approaches to assess the safety, efficacy, quality and performance of FDA-regulated products.” The FDA has stated that new manufacturing technologies are one of their regulatory science priority areas within their strategic plan [5] and it has also created committees for cross-agency talk [6] as well as updated FDA guidance documents on the topic of additive manufacturing [7].

As the field of additive manufacturing is increasingly used to develop medical technologies, the scientific community must conform novel methodologies and unique therapies into the current regulatory framework. In some instances, new products will flow logically into the regulatory schema commensurate with the change in the technology [2]. Often, however, additive manufacturing presents novel content that is unable to fit any existing regulatory pathways and new routes must be forged for the regulatory agency to adequately evaluate the safety and effectiveness of the products. One example is that the FDA requires conventional validation of mechanical performance, which may not be practical or sustainable for customized additive manufacturing devices used for precision medicine. Alternatives for testing include finite-element analysis and computational fluid dynamics, which can serve as useful surrogates but utilize assumptions (i.e., material properties, numerical approximations, boundary conditions, etc.) that have to be verified against known analytical or experimental data to be accepted by the FDA [2,8,9].

The Regulatory Science to Advance Precision Medicine Working Group identified critical topics with strong regulatory science issues to discuss at the Fall Forum. On September 27, 2017, a group of experts convened in Washington, DC, for a Regulatory Science to Advance Precision Medicine workshop on this additive manufacturing topic. (An additional topic, *Omics in Precision Medicine*, was also discussed and will be the subject of a partner publication.) This review summarizes the outcomes of the workshop. We provide a brief overview of the current state of the field of additive manufacturing of medical devices and medical products and the associated regulatory considerations. A description of the regulatory science and educational gaps, potential solutions to those gaps, and ideas for growth of active partnerships with key stakeholders are addressed.

## State of the Emerging Science for Additive Manufacturing

John Fisher, PhD from the University of Maryland laid the foundation of the meeting by providing an overview of the state of the emerging science. The last three decades have seen a 26% growth in medically related additive manufacturing and it now represents a $5 billion industry in the United States [1,10]. Applications range from the manufacture of nonbiodegradable tissue models and implants to “bioprinting,” which refers to the deposition of cells within a bio-ink for tissue regeneration [11].

### Applications of 3D Printed Tissue Models and Medical Implants

Tissue models created by additive manufacturing are becoming essential tools in medical education for demonstrating both normal and pathological anatomies, planning intricate surgeries, and using as imaging phantoms. There is also growing demand for 3D-printed nonbiodegradable implants for dental restoration and wearable prostheses (e.g., nose and ear) for esthetic outcomes [12,13]. In addition, there is active research in the development of biodegradable scaffolds to guide regeneration of damaged and diseased tissues (reviewed in [14,15]). These devices provide unique regulatory and standardization challenges given their potential to be manufactured in very small quantities and tailored to the needs of individual patients. Owing to its high spatial resolution, additive manufacturing is also being used to design delivery modalities that provide spatiotemporal control over drug release profiles [16,17].

### Challenges in 3D Bioprinting

Although additive manufacturing is a decade-old technology, bioprinting remains very much in its infancy [11]. The bulk of innovative research in this space focuses on the development of biocompatible “*inks*” with the appropriate blend of physical properties (that facilitate suspension of cells, extrusion, and fast polymerization in response to light or temperature changes) and biological characteristics (for promoting cell viability, proliferation, differentiation, and tissue formation). These include decellularized extracellular matrix bio-inks derived from various tissues [18,19], which are processed to maintain growth factors and cell-binding proteins to promote cell survival and tissue-specific differentiation. Continued advances include improved printing properties (accuracy, speed, and resolution) using coaxial extrusion [20] and using multiple materials [14]. Ongoing studies are exploring the establishment of spatial gradients that can mimic the structure of composite tissues [21,22], temporal gradients that enable “growth” over time [11,23], and application of augmented reality to collect and process data to further optimize individual therapies [24]. These remarkable advances belie the number of technical and regulatory considerations facing the therapeutic application of bioprinting strategies [25].

## Regulatory Considerations for Additive Manufacturing

Richard McFarland, MD, PhD from the Advanced Regenerative Manufacturing Institute presented an overview of existing FDA regulations, highlighting the tension inherent in simultaneously protecting and advancing public health: protection requires enforcement of consistent regulations, while advancement necessitates the development of new regulatory strategies to accommodate the rapidly changing technological and scientific landscape.

McFarland identified the critical question for the additive manufacturing space: “What is the regulated article?” Is it the 3D printer (hardware and/or software), the “ink” (that might be another medical device or cell-based product), or the final printed object? A review of statutory definitions for medical devices, biologics, and combination products identified no fewer than four possible regulatory pathways to licensing, clearance, or approval for any 3D printer or printed product. Given these distinctions, McFarland advised the audience to prioritize regulatory science opportunities by identifying those “that affect the largest number of potential products,” keeping in mind the projected time to viability of current scientific advancements.

In December 2017, the FDA published new draft and final guidance, which provided direction for cell and tissue-based products that should inform materials selection and study design of 3D-printed products, in particular for homologous use. The document, “Technical Considerations for Additive Manufactured Devices,” while limited in its scope and application, addressed regulatory classification defining common terms such as “custom device” versus “patient-specific device,” and citing appropriate consensus standards for manufacturing both “standard-sized” and “patient-matched” designs [7].

## Addressing Regulatory Science Gaps in Additive Manufacturing as Medical Devices

Advances within the additive manufacturing life sciences field have led to research and production of viable tissues and organs [26,27] and an increased variety of materials, low-cost machines, and potential for new therapeutic application. Several areas of concern to both scientists and regulators were discussed during the Fall Forum breakout session.

### Quality

Quality system elements could be incorporated into the design of the research in real time, which could expedite a medical product’s development lifecycle. Different quality system and manufacturing regulations as well as significantly diverse industry practices for drug, medical device, biologic, or cell and tissue-based products highlight the importance of identifying FDA classification early in the design and development process. An area of particular interest that was noted during the Fall Forum was the governance of the bioprinting products at various stages of development. Several challenging factors remain in designing a set of governing principles to regulate process control, materials, sterility, cell viability, cell size and morphology, reproducibility and speed [21,22].

#### Recommendations

Although additive manufacturing technologies and their applications vary tremendously from medical device to bioprinting, there are common steps in each process that can be assessed in a quality system. Two common themes are highlighted. First, acceptance criteria for raw materials must be considered. For polymeric devices, this may be the molecular weight, thermal characteristics, and viscosity of the polymer. For bioprinting, this may be cellular markers and metabolic assays to assess cellular quality. Second, assessment of variability of the product, likely involving a calibration standard for batch-to-batch comparisons must be determined. For devices, this would include nondestructive microcomputed tomography scanning to assess geometric and printing density metrics. Variability in the printed product would also need to be assessed and acceptance criteria established. This would involve both a calibration standard for batch comparisons as well as a standard for the specific device that is being printed. For bioprinted products, continuous nondestructive monitoring of cell viability and phenotype is critical to applications where the cells are in limited supply, labile, and need to retain higher-order cellular functions [28]. Guidance on relevant parameters such as real-time optical tracking, adhesion force, cell activity indicators, and applicable endpoints will further refine this arena.

### Barriers to a Cohesive Set of Additive Manufacturing Regulations

Perhaps the most significant barrier is that additive manufacturing will be used in several areas of healthcare, including devices, drugs, point-of-care therapies, and biological, cellular, and/or tissue-based products and, therefore, different regulatory pathways may evolve. Although most currently cleared or approved additive manufactured products are medical devices, the high demand for cell and tissue-based products will require a learning curve among scientists from various disciplines who may be unfamiliar with the technical aspects unique to medical device or combination product regulatory pathways. The drastically different regulations that govern these tangentially related but technically disparate areas will require a significant amount of cross-talk between the Center for Biologics Evaluation and Research (CBER) and the Center for Devices and Radiological Health.

#### Recommendations

Translating the usual “best fit” model for medical product regulations to an area of research that embodies highly tailored and patient-specific processes will continue to present a great challenge to both scientists and regulators. In addition, understanding the boundaries of responsibility will be critical to regulators given that some additive manufactured products will be parts in a larger ecosystem. Thus, it may not always be clear when asking if a product is a medical device. Basic scientific aspects of regulation will stay the same (e.g., biocompatibility, electrical testing), but the regulatory challenges will center on knowing who is the manufacturer, how is the product produced, who should take action if problems arise, or software updates. Although review divisions within CBER strive to have a flexible and accommodating review style to meet the rapidly changing products under their purview, sponsors will need to fully understand the boundaries of their role, especially if a sponsor is only one part of a medical product’s lifecycle. With the myriad different additive manufacturing product types, development of clearer product classifications for product types could allow for more thorough guidance documents that address each subset of products.

### Education as a Critical Gap in the Expansion of Additive Manufacturing Expansion

An additional theme was the deficit of additive manufacturing education programs in the United States. Formal education will propel the transformative impact of additive manufacturing in healthcare. A basic understanding of additive manufacturing uses, rules, capabilities, and limitations through training and enhanced technical knowledge will impact those currently working with this technology, as well as students and clinicians entering this field. Currently, educational resources are limited to FDA webinars and FDA guidance documents. There are few accredited higher education programs that offer additive manufacturing curricula in the United States.

#### Recommendations

Large companies such as General Electric have addressed the lack of additive manufacturing education by investing resources into schools to develop education pipelines to train future additive manufacturing talent [29]. Encouraging more companies through incentives to partner and establish these education initiatives would make a tremendous impact toward advancing the field of additive manufacturing while building a skilled and knowledgeable workforce. There is a specific need to establish a bioprinting curriculum that incorporates engineering, biology, regulatory science, quality control, Good Practices (GxP), FDA regulations, process development, manufacturing models, and overall project management. Conversely, the additive manufacturing field is opening up the options for training surgeons with realistic models of healthy and abnormal anatomy [30]. Live training opportunities should be available for technicians using the equipment as well as clinicians who want to incorporate additive manufacturing processes into patient care.

In 2014, a set of regulatory science core competencies was developed to cover the vast array of topics and training needs that fall under the regulatory science umbrella. Included was a plan for tripartite training to provide a deeper understanding of the current regulations and opportunities to evaluate and address new regulatory science challenges in industry, academia, and within the agency [31] and how that is relevant to novel technologies. Through strategic partnerships that include both training and research initiatives, we can bridge the gaps by providing a holistic view through collaboration among scientists, educators, regulators, industry, and clinicians.

## Roles for Active Partnerships

There are several existing public-private-partnerships (PPP) that focus on aspects of biofabrication including additive manufacturing. BioFab USA is one example that began as Manufacturing Innovation Institutes [32] and is now continuing as the Advanced Regenerative Manufacturing Institute [33]. The National Institute for Innovation in Manufacturing Biopharmaceuticals (NIMBL) is another PPP designed to accelerate biopharmaceutical manufacturing innovation and support the development of standards in this area [34]. NIMBL is embracing an educational focus to help in training the biopharmaceutical manufacturing workforce. For these efforts, NIMBL has created a national network to support research, development and training in biomanufacturing. In addition to the General Electric program described earlier, other educational PPP are vital to address these training gaps. This could include collaborations between the CTSA network of academic institutions, the PhRMA Foundation, NIMBL, and the Reagan Udall Foundation for the FDA as well as scientific and engineering associations. All of the recommendations included in this paper would benefit from interactions between biofabrication companies, academic centers, and the key regulatory, research, and standard-setting agencies.

## Conclusion and Recommendations

Additive manufacturing incorporates research from different disciplines and entities with unique objectives that will drive innovative public health advances while also presenting greater challenges to the current regulatory framework. With additive manufacturing, one process can readily be used to produce multiple devices on a daily basis, providing tremendous flexibility into the device design process but also significant variability. It is this unique combination of flexibility and variability that is likely to present the greatest regulatory science challenges.

Finally, innovators of additive manufactured medical devices in both industry and academia must be encouraged to pursue early and frequent engagement with FDA, not only to obtain the most accurate and timely guidance but also to underscore the need for validation of standards and appropriate regulation. Partnerships between sponsors and FDA that leverage technology to incorporate real-world evidence may facilitate FDA’s review and assessment of a product while allowing sponsors to respond and address manufacturing challenges. Increased funding for education in emerging technologies in addition to manufacturing and patient demand for access to personalized manufactured medical devices will inevitably be the key drivers in the marketplace.
